# Nature-themed video intervention may improve cardiovascular safety of psilocybin-assisted therapy for alcohol use disorder

**DOI:** 10.3389/fpsyt.2023.1215972

**Published:** 2023-09-18

**Authors:** Keith G. Heinzerling, Karina Sergi, Micah Linton, Rhianna Rich, Brittany Youssef, Inez Bentancourt, Jennifer Bramen, Prabha Siddarth, Louie Schwartzberg, Daniel F. Kelly

**Affiliations:** ^1^Treatment and Research In Psychedelics (TRIP) Center, Pacific Neuroscience Institute at Providence Saint John’s Health Center, Santa Monica, CA, United States; ^2^Brain Health Center, Pacific Neuroscience Institute at Providence Saint John’s Health Center, Santa Monica, CA, United States; ^3^Moving Art, Los Angeles, CA, United States

**Keywords:** psilocybin, psychedelic-assisted therapy, set and setting, alcohol use disorder, video interventions, nature therapy

## Abstract

**Introduction:**

Psychedelic-assisted therapy with psilocybin has shown promise in Phase 2 trials for alcohol use disorder (AUD). Set and setting, particularly factors facilitating a connection with nature, may positively influence the psychedelic experience and therapeutic outcomes. But to date, randomized controlled trials of interventions to enhance set and setting for psychedelic-assisted therapy are lacking.

**Methods:**

This was a pilot randomized, controlled trial of Visual Healing, a nature-themed video intervention to optimize set and setting, versus Standard set and setting procedures with two open-label psilocybin 25 mg dosing sessions among 20 participants with AUD. For the first session, participants randomized to Visual Healing viewed nature-themed videos during the preparation session and the “ascent” and “descent” phases of the psilocybin dosing session while participants randomized to the Standard condition completed a meditation during the preparatory session and wore eyeshades and listened to a music playlist throughout the dosing session. For the second session 4 weeks later, participants chose either Visual Healing or Standard procedures. Primary outcomes were feasibility, safety, and tolerability of Visual Healing. Secondary and exploratory outcomes were changes in alcohol use, psychedelic effects, anxiety and stress.

**Results:**

Nineteen of 20 (95%) randomized participants (mean age 49 ± 11 years, 60% female) completed the 14-week study. During the first psilocybin session, participants viewed an average of 37.9 min of the 42-min video and there were no video-related adverse events. Peak increase in post-psilocybin blood pressure was significantly less for participants randomly assigned to Visual Healing compared to Standard procedures. Alcohol use decreased significantly in both Visual Healing and Standard groups and psychedelic effects, stress, and anxiety were similar between groups.

**Discussion:**

In this open-label pilot study, viewing Visual Healing videos during preparation and psilocybin dosing sessions was feasible, safe, and well-tolerated among participants with AUD. Preliminary findings suggest that Visual Healing has potential to reduce the cardiovascular risks of psychedelic therapy, without interfering with the psychedelic experience or alcohol-related treatment outcomes. Studies to replicate our findings as well as studies of different set and setting interventions with other psychedelic medications and indications are warranted.

## 1. Introduction

Psychedelic-assisted therapy is an innovative and groundbreaking approach that has the potential to revolutionize the treatment of behavioral, mental health, and substance use disorders (1). One of the earliest examples of psychedelic therapy comes from Canada in the 1950s when Humphry Osmond and Abram Hoffer used mescaline and lysergic acid diethylamide (LSD) to treat alcohol use disorder (AUD) (2). Initially Osmond and Hoffer hypothesized that LSD might treat alcohol problems by triggering a psychotic experience similar to Delirium Tremens thereby motivating the patient to quit drinking to avoid future negative experiences. But they revised this when patients reported a newfound commitment to alcohol abstinence that they attributed to a psychedelic-induced mystical experience which they noted was similar to anecdotal reports of increased motivation to abstain following epiphanies achieved *via* the 12 Steps of Alcoholics Anonymous (3). Following up on Osmond and Hoffer’s experience, a meta-analysis of six randomized controlled trials of LSD for AUD from 1966–70 found statistically significant reductions in alcohol misuse symptoms at three and 6 months but not 12 months and increased odds of sustained alcohol abstinence at three but not 6 months following a single dose of LSD (4). The meta-analysis estimated a number needed to treat (NNT) of 7 for a single dose of LSD to prevent return to drinking in 3 months compared to NNT of 33 for daily naltrexone and 9 for daily acamprosate, two FDA-approved AUD medications. Unfortunately, barriers to clinical research with psychedelics prevented studies from following up on these initial promising results until recently when a randomized, double-blind, placebo-controlled trial in 93 participants with AUD found significantly greater reductions in percent of heavy drinking days, the primary outcome, for psilocybin compared to an active placebo diphenhydramine, when combined with 12 weeks of AUD counseling (5). Promising early studies with LSD along with the recent positive trial of psilocybin for AUD suggest that additional studies of psychedelic therapies for AUD, including psilocybin, are warranted.

A large body of anecdotal experience and clinical studies show that a user’s state of mind (set) and the setting for the psychedelic experience have a strong influence on the quality of the experience and subsequent post-experience outcomes. Hartogsohn describes set as the “personality, preparation, expectation, and intention of the person having the experience,” and setting as the “physical, social, and cultural environment in which the experience takes place” (6). The importance of set and setting in shaping a psychedelic experience were first recognized by indigenous shamans and healers in their ritual and ceremonial use of psychedelics (6). Modern research has identified elements of set and setting including a negative mindset, lack of preparation, major recent life event, no psychological support, and uncomfortable physical surroundings, that are associated with a difficult, challenging, or even harmful psychedelic experience (7). In contrast, having an intention for the psychedelic experience to connect spiritually and/or with nature predicted a more complete mystical experience and greater increases in post-psychedelic wellbeing (8). The intensity of the mystical experience, even when challenging or difficult, is associated with greater post-psychedelic reductions in depression, anxiety, and substance abuse symptoms (9, 10). In light of these findings, modern psychedelic-assisted therapy trials now include standard procedures aimed at creating a set and setting that may facilitate a mystical, therapeutic, and safe experience, including preparing the participant prior to the dosing session, support from experienced therapists sometimes known as guides or facilitators, and a calming physical environment where the participant can recline on a couch wearing eyeshades while listening to specially selected music. In addition to standard set and setting procedures, interventions to augment or enhance standard psychedelic therapy set and setting procedures may increase the chance of a mystical experience, reduce adverse events, and improve clinical outcomes of psychedelic therapies.

**Figure 1:**
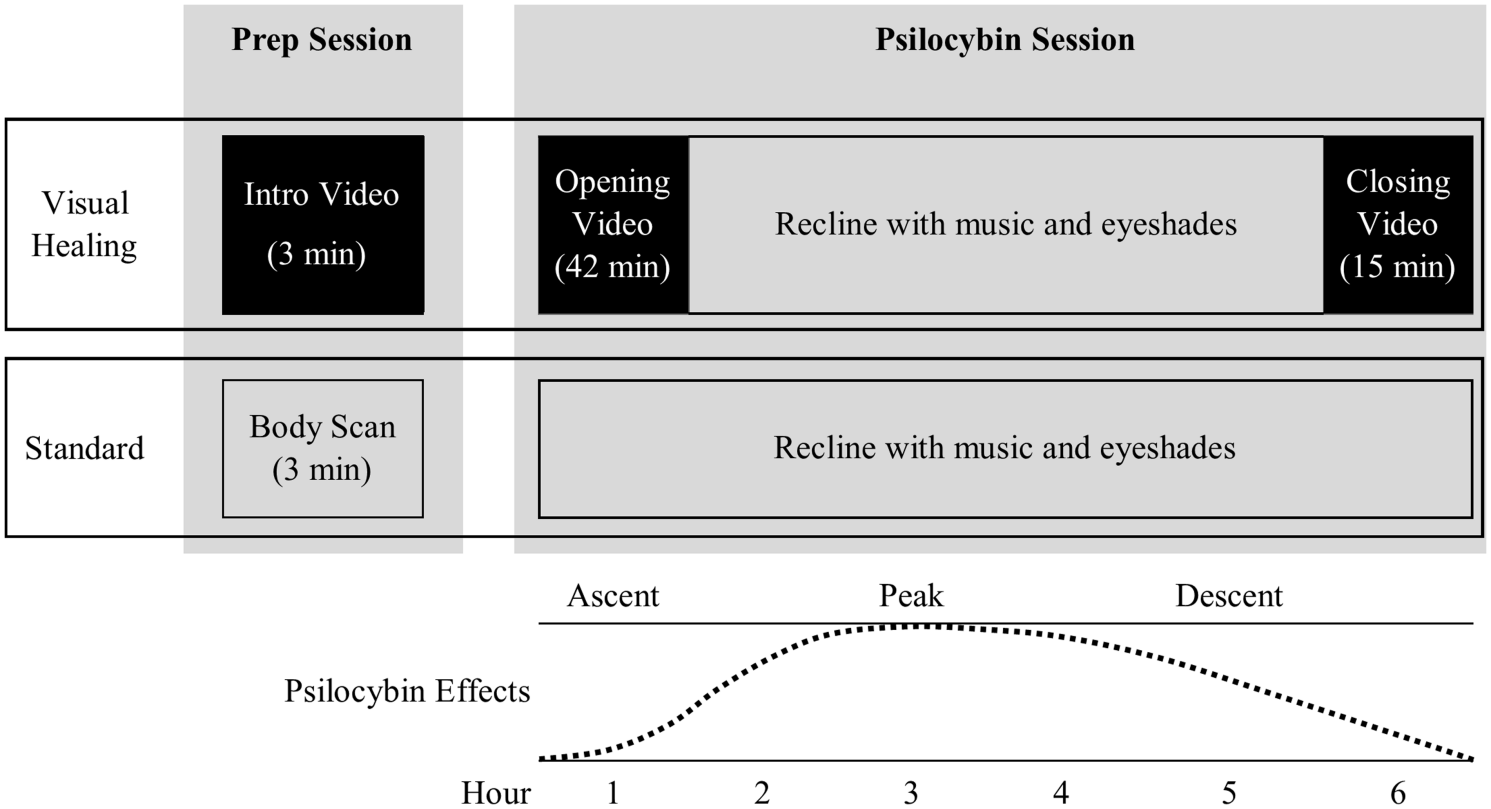
Overview of Visual Healing intervention versus Standard control procedures during the preparatory counseling and psilocybin dosing sessions. * Weeks 2 and 3 participants were randomly assigned to Visual Healing versus Standard procedures while Weeks 6 and 7 participants chose Visual Healing or Standard procedures.

Combining psychedelics with video and virtual reality interventions has been proposed as a means of enhancing or augmenting set and setting for psychedelic therapy (11). However, we are unaware of randomized controlled trials that have assessed the safety and feasibility of integrating video interventions within psychedelic-assisted therapy dosing sessions. Therefore, we performed a pilot randomized, controlled trial to assess the safety and feasibility of Visual Healing, a nature-themed video intervention implemented during the psilocybin dosing session designed to optimize set and setting in psilocybin-assisted therapy for AUD (Figure 1). Standard set and setting procedures in psychedelic clinical trials involve participants reclining on a couch, wearing eyeshades, and listening to music, with participants instructed to direct their attention internally to the psychedelic experience and whether inclusion of a video intervention within the dosing session would be tolerable or interfere with the psychedelic/mystical experience is not known. We hypothesized that the nature-themed Visual Healing might improve psychedelic therapy by reducing participants’ anxiety and apprehension and fostering an intention to connect with nature thereby reducing the risk of a challenging experience or adverse events and increasing the likelihood of a mystical experience and positive therapeutic outcomes. We chose a nature theme for the video as use of psychedelics in traditional healing rituals and outside of clinical trials often occurred in natural and outdoor settings (12) and a greater feeling of connectedness to oneself, others, and the natural world is consistently reported following psychedelic experiences (13–16). E.O Wilson’s *biophilia* hypothesis proposes humans are evolutionarily predisposed to seek connections with nature and other living things (17) and increased urbanization and disconnection from the natural world has been hypothesized to contribute to increasing depression, anxiety, and substance use disorders (18). Bringing art with nature themes, landscapes, and simulated nature into hospital settings has been shown to positively impact patient outcomes including reducing blood pressure, stress, anxiety, and pain (19–22). Together these studies suggest that a pilot trial of a nature-themed video intervention within psilocybin-assisted therapy for AUD is warranted.

## 2. Methods

The study was a pilot randomized, controlled trial of Visual Healing, a nature-themed video intervention to optimize psychedelic-assisted therapy set and setting, versus standard set and setting procedures with open-label psilocybin among 20 participants with AUD. The sample size of 20 participants each undergoing two psilocybin dosing sessions for a total of 40 dosing sessions was deemed to be a sufficient sample size to assess the feasibility of recruiting and enrolling participants in such a trial and was within the budgetary constraints for a pilot trial at our research center. Synthetic psilocybin in 25 mg capsules were provided for the trial by the Usona Institute Investigational Drug Supply Program. The study was performed under a Research IND from the US FDA and Schedule I Researcher Registration from the US DEA held by the Principal Investigator (KH). The study was approved by the Providence Health System IRB, the Research Advisory Panel of California, and was listed on ClinicalTrials.gov (NCT04410913). All study activities were performed at the Pacific Treatment & Research in Psychedelics Program clinic in Santa Monica, California.

### 2.1. Participants

Adults interested in reducing or stopping alcohol use were recruited *via* fliers, ads on local public radio, outreach to doctors, therapists, and alcohol treatment programs, and the research center website.^1^ Interested potential participants underwent an initial telephone screen and those still eligible after the telephone screen were scheduled to meet with the Principal Investigator and complete informed consent procedures at the research clinic.

Eligible participants were age 18 or older; able to read, speak, and understand English; meet DSM-5 criteria for alcohol use disorder, moderate–severe assessed *via* the Structured Clinical Interview for DSM-5 (SCID); report at least 4 heavy drinking days (5 or more alcoholic drinks for males or 4 or more alcoholic drinks for females on the same occasion) in the past 30 days; interested in stopping or reducing alcohol use; willing to practice an effective means of birth control throughout the duration of the study (for women of childbearing potential); able to identify a support person to drive them home after dosing sessions. Participants were not eligible if he/she met any of the following: required medical intervention for alcohol withdrawal, i.e., Clinical Institute Withdrawal Assessment- Alcohol revised (CIWA-Ar) score greater than 7 with a negative (<0.02%) alcohol breath test (referral for alcohol detoxification and rescreen within 30 days was allowed); women who were pregnant or nursing; unwilling/unable to discontinue outside alcohol treatments except self-help; current Major Depressive Disorder or Generalized Anxiety Disorder; use of serotonergic medications, opioids, or benzodiazepines; psychedelic use (not including ketamine) in the past 12 months or > 25 times lifetime; clinically significant cardiac, hepatic, neurological, or endocrine disease including uncontrolled hypertension; personal history or first-degree relative with schizophrenia spectrum or other psychotic disorders (except substance/medication-induced or due to another medical condition), or Bipolar I Disorder; nicotine or drug use disorder; active suicidal ideation with at least some intent to act in the past month or any suicidal behavior in the past 12 months assessed *via* the Columbia-Suicide Severity Rating Scale (C-SSRS).

Use of sleep medications (antihistamine, temazepam at bedtime) was allowed except the night before dosing and participants were allowed to continue non-psychiatric medications such as levothyroxine and antihypertensives. Participants taking medications that could be used to treat alcohol use disorder such as naltrexone or gabapentin discontinued these prior to the study, although only one participant was taking naltrexone (low dose naltrexone for arthritis) during screening.

### 2.2. Procedures

**Figure 2:**
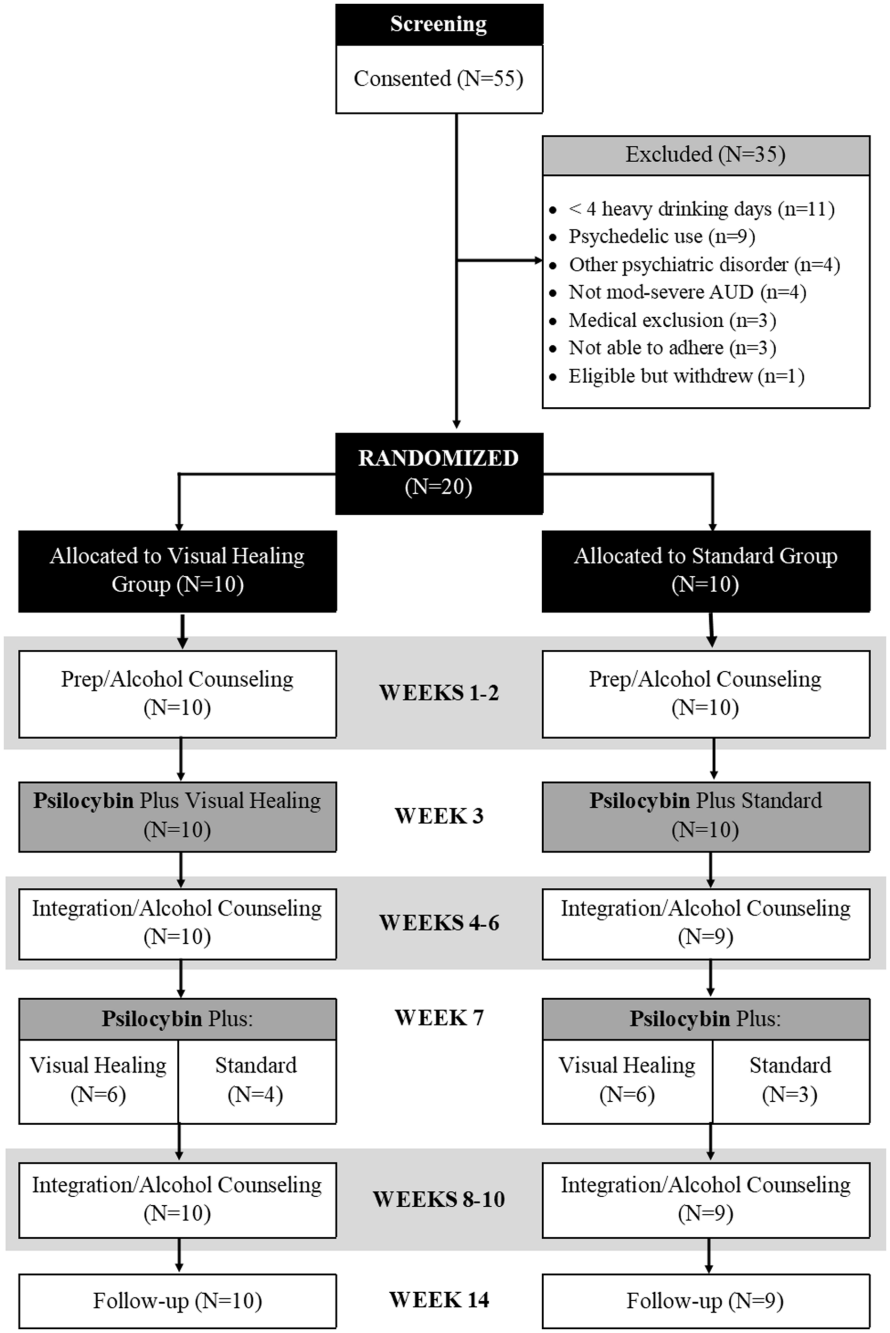
CONSORT Diagram.

After completing baseline and screening assessments, eligible participants in both the Visual Healing and Standard groups attended the research clinic weekly from Weeks 1 through 10 for counseling visits and to complete assessments (Figure 2). In addition, participants underwent two open-label psilocybin dosing sessions 4 weeks apart during Week 3 and Week 7. For the Week 3 dosing session, participants received either the Standard set and setting procedures or the Visual Healing intervention based on random assignment prior to Week 1. For the Week 7 dosing session, participants in both groups chose whether to receive the Visual Healing or Standard procedures. Participants were informed that they would be able to choose to view the video or not for the second psilocybin session during the consent process and reminded again during preparation for each of the dosing sessions. After completion of counseling in Week 10, participants returned to the clinic in Week 14 for a follow-up assessment.

#### 2.2.1. Screening and randomization

Participants met with the Principal Investigator (KH) at the research clinic to complete the informed consent process. Consented participants then underwent a battery of medical and psychological assessments including medical history, physical exam, lab tests, urine drug screen and if indicated pregnancy test, EKG, SCID, C-SSRS, and assessment of past 30-day alcohol use *via* timeline followback and Patient-Reported Outcomes Measurement Information System (PROMIS) Alcohol Use Short Form to assess baseline status and eligibility for the trial. During screening, participants were advised that a breathalyzer negative for alcohol and no significant alcohol withdrawal symptoms on the morning of the psilocybin dosing session was required. Participants then underwent alcohol breathalyzer testing and assessment of alcohol withdrawal *via* the CIWA-Ar and participants unable to abstain from alcohol and/or with alcohol withdrawal symptoms requiring medical intervention were referred for alcohol detoxification treatment. Eligible participants requiring alcohol detoxification were eligible to rescreen following completion of detoxification.

Participants meeting all eligibility criteria were randomly assigned to the Visual Healing intervention (*N* = 10) or Standard set and setting procedures (*N* = 10) and were scheduled for the Week 1 visit to begin study counseling and assessments. Randomization was done *via* a randomization list that was generated prior to the study and maintained by a staff member without direct contact with study participants.

#### 2.2.2. Visual Healing intervention

**Figure 3:**
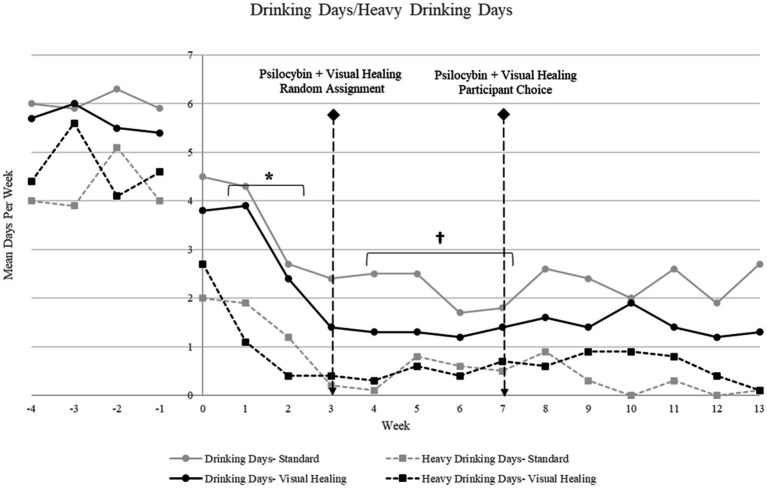
Mean drinking days and mean heavy drinking days per week for participants randomly assigned to the Visual Healing and Standard groups (Weeks −4 through −1: baseline/screening; Weeks 1–2: Preparatory/Alcohol counseling; Week 3 = psilocybin 25 mg plus Visual Healing or Standard procedures by random assignment; Weeks 4–6: Integration/Alcohol counseling; Week 7: psilocybin 25 mg plus Visual Healing or Standard procedures by participant choice; Weeks 8–10: Integration/Alcohol counseling; Weeks 10–14: Follow-up). ^*^ *p* < 0.05 for Weeks 1–2 versus baseline for drinking days and heavy drinking days in both Visual Healing and Standard, ^†^*p* < 0.05 for Weeks 4–7 versus Weeks 1–2 baseline for drinking days and heavy drinking days in both Visual Healing and Standard.

The Visual Healing intervention consisted of three videos created specifically for the trial by one of the authors (LS) in collaboration with co-authors KGH and DFK: (1) a short 3- min Introduction Video shown to participants at the conclusion of each pre-dosing preparatory (prep) counseling session to introduce participants to and prepare them for the intervention and familiarize them with the video format prior to the psilocybin session, (2) a 42- min “Opening Video” played at the start of the psilocybin session (beginning immediately after psilocybin dosing) during the Ascent phase of the psychedelic subjective effects (Figure 1), and (3) a 15- min “Closing Video” played following resolution of the Peak psilocybin effects and during the Descent/Return phase of the session (approximately 4–5 h post-dosing). Similar to the design of music for psychedelic sessions, the Opening and Closing Videos were designed to fit with the typical time course of psilocybin subjective effects and specifically avoided the time when Peak subjective effects occur to avoid external distractions and allow participants to wear eyeshades, listen to music, and focus inward. The videos combined time-lapse, slow motion, and aerial cinematography of nature scenes (e.g., slow motion of a flower blooming or fungi germinating, aerial views of rivers and snowcapped mountains) accompanied by music and no narration with the tone and tempo of the Opening Video slowly and gently building and unfolding to support the psilocybin Ascent experience. The Closing Video included scenes of nature intended to be welcoming and comforting (e.g., a mother and baby whale swimming together). The videos were played on an 85-inch-high-definition LCD television with surround sound stereo speakers in the psychedelic dosing room positioned to allow participants to recline comfortably on the dosing room couch while watching the videos (Supplementary Figure S1).

#### 2.2.3. Counseling sessions

Participants in both the Visual Healing and Standard groups visited the research clinic weekly during Weeks 1 through 10 for 60-min counseling sessions and to complete assessments. Counseling was delivered by a two-person clinician/psychedelic facilitator team consisting of one physician and one therapist. Counseling for the trial combined set and setting procedures typical of psychedelic-assisted therapy including pre-dosing prep sessions and post-dosing integration sessions combined with alcohol use counseling modeled after that provided in clinical trials of medications for AUD (23).

Clinicians were trained prior to the trial and followed a checklist of topics and procedures to be completed in each counseling session to standardize counseling content. Week 1 and 2 sessions were prep sessions that provided participants with information to prepare them for the Week 3 psilocybin dosing session. The objectives of the preparatory sessions were (1) to introduce the participant to psychedelic therapy and prepare them for the experience of the psychedelic dosing session, (2) to provide the therapists with background on the participant’s psychological and psychosocial background and a thorough history of their alcohol use, (3) to build rapport between the therapists and the participant, and (4) assist the participant in identifying an intention for their upcoming psychedelic dosing session. Clinicians told participants that the objective of the treatment was to assist him/her in stopping or reducing their alcohol use but a commitment to a long-term goal of alcohol abstinence was not required. Participants were reminded that sufficient abstinence to achieve a negative breathalyzer and no alcohol withdrawal symptoms on the psilocybin dosing day was required for dosing and abstinence leading up to the dosing sessions was recommended as a means of insuring safety as well as a clear mind for the psilocybin dosing sessions. In discussing the potential role of the psychedelic sessions in addressing their alcohol problems, participants were encouraged to use the psychedelic experience to “explore, examine, and potentially transform, their relationship with alcohol.”

During these prep sessions, participants consented or declined to allow clinicians to provide “supportive touch,” such as holding a hand or placing a hand on the shoulder. At the conclusion of the Week 2 session, clinicians encouraged participants to keep a mindful state during the upcoming dosing session, to remain “open to the experience,” and avoid resistance as a means of achieving of a favorable therapeutic result. Clinicians then instructed participants that focusing on their breathing was a tool they could use during the dosing session if the experience seemed too intense or frightening or if they became anxious and participants randomized to the Standard group were led by the clinicians in completing a 3-min body scan guided meditation using a free online meditation resource.^2^ Participants randomized to the Visual Healing group received the same advice about breathing but viewed the 3-min Visual Healing Intro Video in place of the guided body scan mediation. Participants were then scheduled for the Week 3 psilocybin dosing session.

The day after the Week 3 dosing session, or occasionally 2–3 days after, and then weekly from Weeks 4 through 10, participants returned to the clinic to complete questionnaires and assessments and integration counseling sessions with the two clinicians. The objectives of the integration sessions were to assist the participant in reflecting on the psychedelic dosing experience and in identifying any new insights, motivations, or experiences that could be used to prompt or inspire a change in emotions, thoughts or drinking behaviors. Clinicians guided the participant in reviewing the psychedelic experience and in developing a plan to change their thoughts and behaviors related to alcohol use or their “relationship with alcohol.” Recent alcohol use was reviewed along with any participation in community self-help groups and the participant’s pre-dosing intention(s) were revisited and updated as needed in light of experience from the dosing session. The approach was non-directive, but as the objective of the treatment was to reduce or stop problematic alcohol use, clinicians recommended the participant make a change in his/her drinking behavior including setting a goal of alcohol abstinence although reductions in alcohol use without abstinence were supported as long as the clinicians did not identify immediate risks for serious harm (e.g., driving after drinking).

Similar to Week 2, at the conclusion of the Week 6 session participants and clinicians again reviewed advice for the Week 7 psilocybin dosing session except that participants in each group chose whether to do the body scan meditation and Standard dosing day procedures or to watch the Visual Healing Intro video and then proceed with the Visual Healing intervention during the dosing session.

#### 2.2.4. Psilocybin dosing sessions

On the morning of the psilocybin dosing session (Weeks 3 and 7) participants underwent pre-dosing safety and eligibility assessments including C-SSRS, vital signs, urine drug test and alcohol breathalyzer which were reviewed by the study PI. Any participant with a positive alcohol breath test, urine drug screen or pregnancy test, significant alcohol withdrawal symptoms (CIMA-Ar > 7), acute suicidality, abnormal blood pressure or heart rate, or other clinically significant issues did not undergo dosing and was able to reschedule the dosing session once. Participants cleared for dosing by the PI were accompanied by the two-clinician team to the dosing room and after getting settled received 25 mg psilocybin orally. At this point, participants who had been randomized to Standard procedures during Week 3 and participants in either group who chose Standard procedures in Week 7 reclined on a couch in the dosing room, put eyeshades on, and listened to a music playlist (Supplementary Table S1) through speakers in the room or optional headphones. Participants who were randomized to Visual Healing in Week 3 and those who chose Visual Healing in Week 7 reclined on the couch and watched the 42-min Opening Video (Figure 1). At the conclusion of the video or if the participant lowered their eyeshades or asked to turn off the video prior to the conclusion, the video was turned off and participants then reclined on the couch with eyeshades and the music playlist was started accounting for the time viewing the video. After resolution of peak psilocybin effects (approximately 4–5 h post-dose) participants were offered the chance to view the 15-min Closing Video if they desired.

Participants remained in the dosing room accompanied at all times by the two-clinician team for at least 7 h from the time of psilocybin ingestion, typically from 9 AM to 4 PM. The study PI (also MD) was present for all dosing sessions. The clinicians minimized interaction with the participant during the session to avoid distracting the participants from the internal psychedelic experience but were available to provide gentle support, such as holding a hand or placing a hand on the shoulder, and encouragement if needed and to monitor the safety of the participant. Blood pressure, heart rate, and adverse events were assessed at 30, 60, 90, 120 min, and 4, 6, and 7 h after psilocybin administration and written procedures and rescue medications (e.g., diazepam, risperidone, clonidine) to address potential adverse events and treatment complications were available to the study physician. At 7 h post-psilocybin, participants completed questionnaires and after clearance for discharge by the study physician, participants were discharged to their pre-determined support person who drove them home.

#### 2.2.5. Follow-up visit

After completion of the final counseling session in Week 10, participants then returned to the clinic to complete end of study safety and follow-up assessments in Week 14.

### 2.3. Study endpoints

The primary objective of this pilot study was to determine the feasibility, safety, and tolerability of adding Visual Healing to psilocybin-assisted therapy among participants with AUD as assessed with the primary endpoints: recruitment and retention rates, minutes of Visual Healing film viewed, adverse events, and vital signs. The secondary objective was to explore whether Visual Healing improves treatment outcomes for AUD more than Standard procedures as assessed by self-reported alcohol use on the Timeline Followback and the PROMIS Alcohol Use Short Form. Exploratory objectives included examining whether (1) Visual Healing during the Prep session reduces pre-dosing session anxiety/apprehension more than standard Prep session procedures assessed by the State Trait Anxiety Inventory (STAI)-SF State Anxiety measure and AM salivary cortisol and/or (2) whether Visual Healing during the psilocybin dosing session alters the psychedelic experience compared to Standard procedures assessed *via* the Mystical Experience Questionnaire (MEQ), Ego Dissolution Inventory (EDI), Emotional Breakthrough Inventory (EBI), Challenging Experience Questionnaire, and Questionnaire for Psychotic Experiences. As the primary aim for this manuscript was to explore the effect of the Visual Healing intervention, analyses are focused on the Week 3 psilocybin session in which participants were randomly assigned to the Visual Healing versus Standard procedures.

### 2.4. Statistical analyses

Treatment groups (Visual Healing vs. Standard) were compared using Kruskal-Wallis tests (continuous variables) or Fisher’s exact tests (categorical variables) on demographic and other measures. Mixed effects repeated measures models were used to compare changes on all outcome measures between groups, with group, time, and the interaction of group by time as predictors. A significant group by time interaction, indicating differential change between groups, was further investigated with follow-up tests to determine the timepoint(s) at which significance was detected. In the absence of a significant group by time interaction, the main effect of time was examined to determine if there was an overall change across both treatment groups. Given that this is a pilot feasibility study, we set the significance level at *p* < 0.05 for analyses.

## 3. Results

Participant flow in the study is shown in Figure 2. Of the 55 participants signing informed consent and entering screening, 20 were deemed eligible and entered into the trial and 35 were excluded due to not meeting alcohol use criteria, past psychedelic use, other psychiatric disorders, and medical reasons. Nineteen of the 20 (95%) randomized participants completed the 14-week study intervention and follow-up period. One participant in the Standard group was discontinued in Week 6 due to an alcohol relapse requiring inpatient alcohol detoxification and treatment.

Participants were predominantly aged between 41 and 60 years of age, more women than men, and the majority were White and not Hispanic (Table 1). There were no significant differences in baseline demographics, alcohol use disorder severity, drinking days, PROMIS Alcohol scores, or anxiety between participants randomized to the Visual Healing versus Standard groups (*p* > 0.05).

**Table 1:** Demographics of participants randomly assigned to the Visual Healing (*N* = 10) versus Standard Procedures (*N* = 10) groups (*p* > 0.05 for all comparisons).

	Visual Healing (*N* = 10)	Standard Procedures (*N* = 10)
Age, mean years (S.D)	46.9 (8.5)	51.0 (13.2)
*Age, N (%)*		
18–30 years	0 (0%)	1 (10%)
31–40 years	2 (20%)	1 (10%)
41–50 years	5 (50%)	2 (20%)
50–60 years	3 (30%)	3 (30%)
> 60 years	0 (0%)	3 (30%)
*Sex, N (%)*		
Male	4 (40%)	4 (40%)
Female	6 (60%)	6 (60%)
*Race, N (%)*		
White	9 (90%)	10 (100%)
Asian & Native Hawaiian or Pacific Islander	1 (10%)	0 (0%)
*Ethnicity, N (%)*		
Hispanic	1 (10%)	1 (10%)
Not Hispanic	9 (90%)	9 (90%)
*Alcohol Use Disorder, N (%)*		
Moderate	2 (20%)	5 (50%)
Severe	8 (80%)	5 (50%)
*Alcohol Use, past 4 weeks, mean (S.D.)*		
Drinking days per week	5.7 (1.9)	6.1 (1.8)
Heavy drinking days per week	4.7 (2.0)	4.4 (2.1)
*PROMIS Alcohol Use score, mean (S.D.)*		
T-Score	57.7 (7.1)	56.1 (8.6)
*State–Trait Anxiety Inventory, mean (S.D.)*		
Total score	40.1 (9.7)	33.7 (8.0)

### 3.1. Tolerability and acceptability of the Visual Healing intervention

#### 3.1.1. Exposure to the Visual Healing intervention

During the Week 3 psilocybin session, participants in the Visual Healing group viewed the 42-min Opening Video for an average of 37.9 min (Table 2) with 4 of the 10 participants viewing the entire opening video while the remaining six participants lowered their eyeshades prior to completion of the video. Six of the 10 participants chose to view the 15-min Closing Video watching an average of 8.3 min. When participants in both groups were offered the choice to view the videos during the Week 7 psilocybin session, 6 of 10 in the Visual Healing group chose to watch the Opening Video again and 5 of the 9 remaining in the Standard group chose to view the video for the first time. Half of the Visual Healing group but only one of the participants in the Standard group chose to watch the Closing Video during week 7. There were no statistically significant differences between the Visual Healing and Standard groups in minutes viewed or percent of participants viewing the videos (*p* > 0.05 for all).

**Table 2:** Exposure to the Visual Healing Intervention during psilocybin sessions among participants by random assignment in Week 3 and by participant choice in Week 7 (Maximum running time 42 min for opening video and 15 min for closing video. *p* > 0.05 for all comparisons between Visual Healing and Standard groups).

	Opening video		Closing video	
	Visual Healing	Standard	Visual Healing	Standard
*Week 3 (Random assignment)*				
Viewed video, % (*N*)	100% (10/10)	N/A	60% (6/10)	N/A
Minutes viewed, Mean ± Standard deviation (Range)	37.9 ± 4.4 (32.0–42.0)	N/A	8.3 ± 7.3 (0.0–15.0)	N/A
*Week 7 (Participant choice)*				
Viewed Video, % (*N*)	60% (6/10)	67% (6/9)	50% (5/10)	11% (1/9)
Minutes viewed, Mean ± Standard deviation (Range)	19.8 ± 18.6 (0.0–42.0)	24.0 ± 19.4 (0.0–42.0)	7.3 ± 7.7 (0.0–15.0)	1.7 ± 5.0 (0.0–15.0)

#### 3.1.2. Adverse events

Adverse events were mild to moderate with headache, elevated blood pressure, and nausea being most frequent (Table 3). The frequency of adverse events reported by participants in the Standard group was higher than for the Visual Healing group but the difference was not statistically significant (Kruskal Wallis test *p* value = 0.07). One participant in the Visual Healing group vomited at the conclusion of the opening video and was unsure if the video or the medication had contributed more to the vomiting but chose to not view the videos in Week 7 and did not vomit. Otherwise, there were no adverse events that were directly related to video viewing.

**Table 3:** Frequency of adverse events rated as possibly, probably, or definitely related to study interventions among participants overall and in the Visual Healing versus Standard groups.

	Overall	Visual Healing	Standard
Headache	16	5	11
Elevated blood pressure	10	3	7
Nausea	8	2	6
Twitching	5	2	3
Anxiety	4	3	1
Feeling flushed	3	3	0
Tachycardia	3	0	3
Abdominal pain	1	0	1
Body aches	1	0	1
Bradycardia	1	1	0
Depressed mood	1	0	1
EKG abnormality	1	1	0
Insomnia	1	0	1
Low blood pressure	1	1	0
Synesthesia	1	0	1
Jaw clenching/pain	1	0	1
Tinnitus	1	0	1
Tremor	1	1	0
Vivid dreams	1	0	1
Vomiting	1	1	0
Wooziness	1	0	1
**TOTAL**	63	23	40

There were two Serious Adverse Events, both of which occurred with the same Standard group participant who required treatment in the Emergency Department for severe alcohol intoxication during Week 2 (prior to any psilocybin sessions) and was admitted to an inpatient alcohol detoxification program in Week 6 following a severe alcohol relapse resulting in study termination due to the need for inpatient care. The participant has a history of multiple prior inpatient alcohol treatment episodes and there was no indication the relapse was due to study participation. No participant received rescue medications during the trial.

#### 3.1.3. Participant satisfaction with the Visual Healing intervention

A majority of participants’ responses agreed or strongly agreed that Visual Healing helped them to feel more relaxed and prepared for the psychedelic session and to feel more connected to nature (Table 4). Twenty six percent (26%) of responses strongly agreed that Visual Healing improved the psychedelic experience and 37% strongly agreed that they would choose Visual Healing for future psychedelic experiences. Seventy four percent (74%) of responses disagreed or strongly disagreed that Visual Healing distracted them from the psychedelic experience.

**Table 4:** Participant rating of satisfaction with the Visual Healing intervention within the psychedelic sessions.

	Strongly disagree	Disagree	Somewhat disagree	Neither agree nor disagree	Somewhat agree	Agree	Strongly agree	N/A
Visual Healing during the prep session helped me feel more relaxed and prepared	4% (1)	0% (0)	4% (1)	7% (2)	22%(6)	22% (6)	37% (10)	4% (1)
Visual Healing during the psychedelic session improved my psychedelic experience	4% (1)	0% (0)	4% (1)	19% (5)	30% (8)	11% (3)	26% (7)	7% (2)
Visual Healing during the psychedelic session distracted me from my psychedelic experience	33% (9)	41% (11)	7% (2)	7% (2)	4% (1)	4% (1)	0% (0)	4% (1)
Viewing Visual Healing helped me to feel more connected to nature	4% (1)	0% (0)	0% (0)	15% (4)	19% (5)	26% (7)	33% (9)	4% (1)
I would choose Visual Healing for my future psychedelic experiences	4% (1)	7% (2)	0% (0)	22% (6)	22% (6)	4% (1)	37% (10)	4%(1)

Responses from 27 sessions for which participants provided feedback (total may exceed 100% due to rounding).

Most comments from participants regarding incorporation of Visual Healing within the psychedelic sessions were positive with some qualifications. Example comments include:

- “[The videos] were helpful to ease into the medicine session but once the effects began, I did not want to continue with the video. However, viewing it at the close of the session was calming & grounding, and helpful to regain a sense of normality.”
- “[The videos] helped ease into the experience and were relaxing. Helped the mind focus less on the clinical setting and more on the upcoming session and the connection with nature/world.”
- “Great experiences overall, especially the second session where I had a better idea of the process and what I could expect. Visual Healing videos helped set the tone and connect with nature. The music played a bigger role than video for me.”

Only one comment was clearly negative: “It was annoying, cliché.”

### 3.2. Clinical outcomes

#### 3.2.1. Alcohol use

Alcohol use decreased significantly in the overall sample from baseline to the Week 1–2 preparatory/alcohol counseling period (mean drinking days per week: *F* (5,88) = 2.74, *p* = 0.02, mean heavy drinking days per week: *F* (5, 88) = 7.08, *p* < 0.001), and decreased again from Weeks 1–2 to Weeks 4–7 after the Week 3 psilocybin session (mean drinking days: *F* (6,107) = 7.16, *p* < 0.0001, mean heavy drinking days per week: *F* (6, 107) = 15.76, *p* < 0.001), and then remained low throughout the remainder of the treatment and follow-up periods. There were no significant differences in alcohol use for participants randomized to the Visual Healing versus Standard groups at baseline or any time point (Figure 3).

For both groups, PROMIS Alcohol Use total score decreased significantly with time (*F* (4, 18) = 12.9, *p* < 0.0001) but there were no between-group differences in the trajectory. Both groups exhibited a significant decrease from baseline to Week 1 (*t* (18) = 3.40, *p* = 0.003) and from Week 1 to post-psilocybin in Week 5 (*t* (18) = 3.61, *p* = 0.002) and then remained lower without any further significant change from Week 5 to Week 9 (*t* (18) = 1.79, *p* = 0.09) and Week 9 to 14 (*t* (18) = 0.25, *p* = 0.81). Responses to individual PROMIS questions regarding problematic drinking behaviors were predominantly “often/always” at baseline and prior to psilocybin dosing and shifted to more “never/rarely” responses following the Week 3 and 7 psilocybin sessions (Supplementary Figure S2).

#### 3.2.2. Cardiovascular response to psilocybin

Pre-psilocybin blood pressure and heart rate did not differ at the start of the Week 3 psilocybin session among participants randomly assigned to the Visual Healing versus Standard groups (*p* > 0.05). Overall, blood pressure and heart rate increased post-psilocybin during the dosing session, but increases were mild–moderate, there were no serious cardiovascular adverse events or complications, and blood pressure and heart rate had returned to normal levels by the end of the seven-hour post-dosing observation period when participants were assessed for discharge.

**Figure 4:**
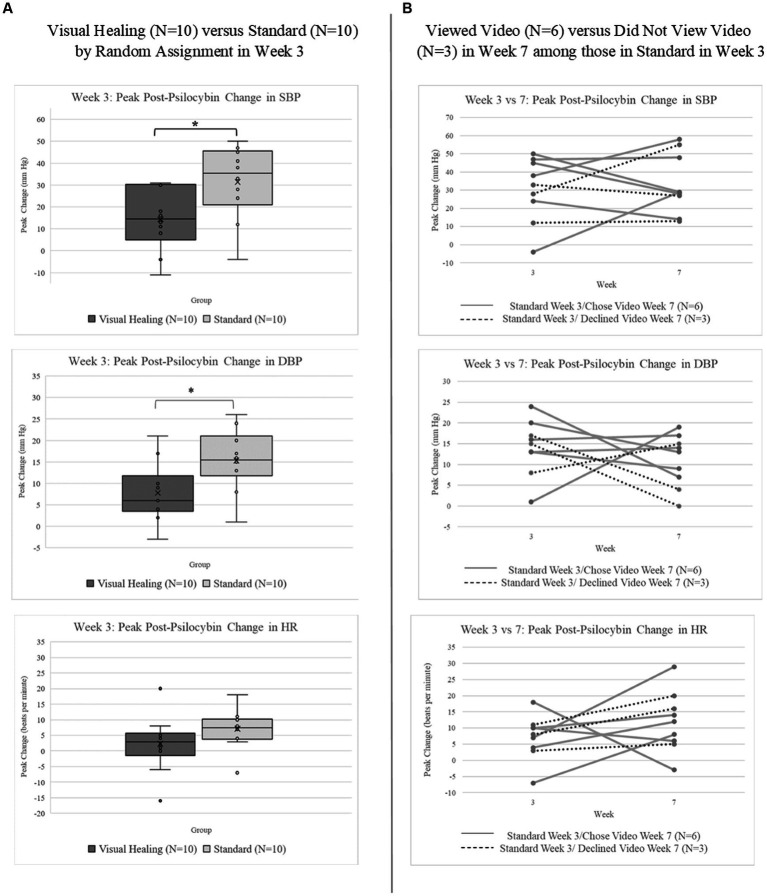
**(A)** Peak change in systolic (SBP) and diastolic blood pressure (DBP) and heart rate (HR) following 25 mg psilocybin administered during the Week 3 psilocybin session among participants randomly assigned to the Visual Healing (*N* = 10) versus Standard group (*N* = 10). ^*^p < 0.05 for Visual Healing versus Standard. **(B)** Peak change in SBP, DBP, and HR following 25 mg psilocybin in Weeks 3 and 7 for individual participants in the Standard group who chose (*N* = 6) versus did not choose (*N* = 3) to view the Visual Healing video in Week 7.

The peak increase in blood pressure pre- to post-psilocybin was significantly less for participants randomly assigned to Visual Healing compared to Standard set and setting (Figure 4A; systolic BP: Kruskal-Wallis test *χ*^2^ (1) = 4.98, *p* = 0.02; diastolic BP: *χ*^2^ (1) = 4.03, *p* = 0.04). Peak increase in heart rate pre- to post-psilocybin was similarly less for Visual Healing participants compared to Standard group, however the difference was not statistically significant (*χ*^2^ (1) = 2.78, *p* = 0.10). The peak change in cardiovascular parameters during the Week 3 versus Week 7 psilocybin sessions among participants in the Standard group (randomly assigned to no video in Week 3) who chose to view the Visual Healing video (*N* = 6) versus declined the video (*N* = 3) in Week 7 is depicted in Figure 4B.

#### 3.2.3. Subjective and psychedelic effects

There were no significant differences in psilocybin subjective effects as assessed *via* the MEQ30, EDI, or EBI during the Week 3 psilocybin session between participants randomly assigned to the visual Healing versus Standard groups (*p* > 0.05 for all comparisons, Table 5). Overall, 50% of participants met criteria for a complete mystical experience, including 40% in the Visual Healing and 60% in the Standard group (Fisher’s exact *p* = 0.7). Around 10% of participants in both groups rated the psilocybin experience as the single most meaningful or spiritually important event in their life while 30% of the Visual Healing group and 44% of the Standard group rated it as among the top 5 most meaningful lifetime experiences (Table 5).

**Table 5:** Subjective psychedelic effects assessed with the Mystical Experience Questionnaire (MEQ-30), Ego Dissolution Inventory, and Emotional Breakthrough Inventory at the conclusion of the Week 3 psilocybin dosing session and the Persisting Effects Short Questionnaire in Week 6 among participants randomly assigned to the Visual Healing versus Standard group.

	Visual Healing	Standard
**MEQ30** (Percent of maximum score)		
Total (Mean ± SD (Range))	0.60 ± 0.26 (0.25–0.96)	0.71 ± 0.26 (0.24–1.00)
Mystical	0.56 ± 0.30 (0.13–1.00)	0.68 ± 0.31 (0.13–1.00)
Positive mood	0.63 ± 0.24 (0.40–1.00)	0.69 ± 0.26 (0.23–1.00)
Transcendence of time and space	0.57 ± 0.29 (0.20–1.00)	0.75 ± 0.23 (0.40–1.00)
Ineffability	0.75 ± 0.27 (0.33–1.00)	0.83 ± 0.20 (0.47–1.00)
Complete mystical experience (% (*N*))^*^	40% (4/10)	60% (6/10)
**Ego dissolution inventory** (Mean ± SD (Range))	5.24 ± 2.85 (1.54–9.79)	6.34 ± 2.48 (0.60–8.91)
**Emotional breakthrough inventory** (Mean ± SD (Range))	6.61 ± 2.49 (2.98–9.78)	7.55 ± 1.59 (4.67–9.68)
**Persisting effects questionnaire** (%, (*N*))		
The psilocybin experience was the single most meaningful experience of my life	10% (1/10)	11% (1/9)
The psilocybin experience was among the top 5 most meaningful experiences of my life	30% (3/10)	44% (4/9)
The psilocybin experience was the single most spiritually significant experience of my life	10% (1/10)	11% (1/9)
The psilocybin experience was among the top 5 most spiritually significant experiences of my life	30% (3/10)	44% (4/9)
The psilocybin experience and contemplation of the experience increased my personal well-being and life satisfaction very much	20% (2/10)	44% (4/9)
Since the experience my behavior has changed in positive ways extremely or strongly	40% (4/10)	56% (5/9)

^*^Complete mystical experience defined as scores on all MEQ30 factors ≥60% of maximum possible score.

The total Challenging Experience Questionnaire (CEQ) score as well as all subscale scores were lower in the Visual Healing group compared to the Standard group but the only between group difference that approached statistical significance was for the Fear subscale (Kruskal-Wallis test *χ*^2^ (1) = 4.00, *p* = 0.05; Supplementary Figure S3).

Scores were zero or near zero for the total and subscale scores of the Questionnaire for Psychotic Experiences in both the Visual Healing and Standard groups (data not shown).

#### 3.2.4. Anxiety and stress

There were no significant between-group differences in changes in mean anxiety scores for participants in the Visual Healing versus Standard groups (*F* (6,18) = 1.15, *p* = 0.4); however there was a significant time main effect (*F* (6,18) = 5.28, *p* = 0.003), indicating that both groups decreased in anxiety with time (Supplementary Figure S4A). Anxiety scores the day after the psilocybin session in Week 3 versus Week 7 among participants in the Standard group (randomly assigned to no video in Week 3) who chose to view the Visual Healing video (*N* = 6) versus declined the video (*N* = 3) in Week 7 are depicted in Supplementary Figure S4B. Further, there were no significant between-group differences in changes in stress assessed with morning salivary cortisol measurement (*F* (2,18)= 1.90, *p* = 0.18) nor was there a significant time main effect (*F* (2, 18) = 2.25, *p* = 0.13; Supplementary Figure S5).

## 4. Discussion

Despite widespread agreement that set and setting have an important influence on psychedelic experience, we are aware of no randomized controlled trials assessing interventions aimed at altering or augmenting set and setting for psychedelic therapy as a potential means of optimizing the psychedelic experience and/or treatment outcomes. We performed a randomized controlled pilot clinical trial of Visual Healing, a nature-themed video intervention, to augment and improve set and setting for psychedelic-assisted therapy in AUD. Participants randomized to Visual Healing during the psilocybin dosing session viewed the majority of the opening video, there were no sessions where the participant or clinicians stopped the video due to interference with the psychedelic therapy, and there were no adverse events clearly related to the video intervention. Recruitment and retention were successful with 95% of participants completing the 14-week study and one participant in the Standard group, who had not been exposed to the Visual Healing intervention, terminating early due to requiring inpatient alcohol treatment. There were large reductions in alcohol use and problematic drinking behaviors in both the Visual Healing and Standard groups and no differences in psychedelic subjective effects or experience between sessions with and without the Visual Healing videos suggesting that the video did not interfere with potential therapeutic effects of psilocybin. Importantly, post-psilocybin increases in blood pressure were lower in participants randomly assigned to the Visual Healing intervention compared to Standard procedures suggesting that Visual Healing my reduce the risk of cardiac complications in psychedelic therapy. Participant satisfaction with inclusion of Visual Healing in the psychedelic dosing session was high with a majority reporting Visual Healing helped them to feel relaxed and prepared for the psychedelic session. Together these results suggest that incorporating Visual Healing into psilocybin-assisted therapy for AUD was feasible and acceptable and may improve safety by reducing risk of cardiovascular complications. Additional studies of Visual Healing and other interventions to augment and enhance set and setting for psychedelic therapy are warranted.

### 4.1. Visual Healing may increase impact of psychedelic therapies by reducing risk of cardiovascular complications

Transient increases in blood pressure and heart rate are typical following administration of psilocybin as well as most medications being tested for psychedelic therapy including MDMA, LSD, and especially ketamine. Cardiostimulant effects of psychedelics may result from direct pharmacologic activation of the sympathetic nervous system or indirectly due to the intense and emotional subjective psychedelic experience (24). While cardiovascular complications have been rare in modern psychedelic clinical trials, the current trial, like the majority of psychedelic trials, had very strict exclusion criteria for any cardiac disease, EKG abnormalities, or hypertension and some have expressed concerns that cardiac safety concerns may limit the utility of psychedelic therapy especially in elderly or frail populations (25). Use of ketamine off-label for depression and other mental health and alcohol use disorders has expanded dramatically and although typical increases in blood pressure and heart rate following sub-anaesthetic doses of ketamine are mild to moderate and transient, concerns about “treatment-emergent” hypertension prevents patients with cardiovascular co-morbidity from potentially benefitting from ketamine therapy and increases the cost of ketamine treatment due to guidelines requiring frequent monitoring of vital signs and availability of rescue medications (26). Post-psilocybin increases in blood pressure were lower in sessions where participants were randomly assigned to Visual Healing. We had hypothesized that viewing the Visual Healing video at the end of the preparatory session and the start of the psilocybin dosing session would reduce anxiety and apprehension. Participants did report that viewing Visual Healing during the initial ascent phase of the psilocybin experience did help them to better “ease into” peak psychedelic effects although there were no significant differences between Visual Healing and Standard groups in anxiety or stress assessed with the STAI or salivary cortisol. Visual inspection of plots comparing cardiovascular and anxiety measures for Week 3 versus Week 7 psilocybin sessions among participants in the Standard group who were randomized to no Visual Healing video during the Week 3 psilocybin session and went on to choose to watch or not watch the video in Week 7 suggest a possible trend towards lower blood pressure and higher heart rate in Week 7 overall but the small number of participants and lack of random assignment to the video precludes making any conclusions regarding the role of choosing the video in Week 7. If confirmed in follow-up studies, Visual Healing and other set and setting interventions may increase the benefits and reach of psychedelic therapies by blunting the post-psychedelic increase in blood pressure thereby reducing the cardiovascular risks.

### 4.2. Alcohol use outcomes for psilocybin with Visual Healing and standard procedures were similar to those seen in prior studies of psychedelics for AUD

In the 1950’s-60’s psychedelic therapies, including psilocybin and LSD, were considered very promising treatments for AUD (27). A meta-analysis of randomized controlled trials of LSD for AUD from the late 1960s found a significant reduction in alcohol misuse following a single dose of LSD (4) but relatively few modern, well controlled clinical trials have assessed psychedelics for treating AUD. Two pilot randomized, controlled trials of ketamine for AUD found ketamine reduced alcohol use more than a saline placebo in one trial (28) and active placebo midazolam in the other (29). In the largest modern clinical trial of a psychedelic for AUD to date, 95 participants with AUD underwent two dosing sessions, with random assignment to psilocybin or diphenhydramine an active placebo, along with psychotherapy for AUD (5). Psilocybin was dosed by weight and a dose increase was allowed for the second session based on the participants preference, MEQ30 scores, and dose-related adverse events with participants receiving on average 28.3 mg psilocybin in the first session and 37.7 mg in the second. Reductions in percentage of heavy drinking days, the primary outcome, were significantly lower for psilocybin compared to diphenhydramine and reductions in percentage of drinking days favored psilocybin but only approached significance (*p* = 0.05). Although the current trial was open-label psilocybin and lacked a placebo group and therefore conclusions regarding the efficacy of psilocybin in reducing alcohol use cannot be made, participants in both the Visual Healing and Standard groups had large reductions in drinking days and heavy drinking days that were sustained throughout the 14-week study period and were similar to those reported by Bogenschutz et al. (5) Furthermore, we observed large reductions in the PROMIS alcohol use score reflecting major reductions in the percentage of participants endorsing compulsive drinking behaviors such as “I had trouble controlling my drinking” and “It was difficult for me to stop drinking after one or two drinks.” The current trial administered a fixed dose of 25 mg psilocybin during both dosing sessions and mean scores on the MEQ 30 for the Visual Healing (0.60 ± 0.26) and Standard (0.71 ± 0.26) groups were similar to mean MEQ 30 scores in Bogenschutz et al. (0.59 ± 0.24 in the first session and 0.64 ± 0.21 for the second higher dose session). Lastly, both trials observed reductions in alcohol use prior to the first psilocybin session during which participants received psychotherapy. In our trial this was by design as participants were required to abstain from alcohol prior to the psilocybin dosing for safety reasons and participants unable to achieve a negative alcohol breathalyzer without clinically significant alcohol withdrawal symptoms during screening were not eligible. Additional studies assessing the efficacy of psilocybin in reducing alcohol use and improving alcohol-related treatment outcomes and comparing results with psilocybin to current AUD treatments such as naltrexone for example are needed.

### 4.3. Limitations and future directions

This study is limited by the open-label psilocybin design, lack of blinding with the video intervention, the small number of participants, and a sample that was primarily White. However, to our knowledge, there are no previous randomized, controlled trials that have assessed video or nature-themed interventions to optimize set and setting for psychedelic-assisted therapy. Consequently, this small pilot trial assessing feasibility, safety, and tolerability of Visual Healing during the psychedelic dosing session was the most appropriate study design. Additional larger studies with more diverse samples adequately powered to detect potential differences in important clinical outcomes are needed prior to any conclusions regarding the utility of Visual Healing or other similar set and setting interventions. The design of the video intervention including the length of the videos and the time within the psilocybin session during which the intervention was deployed were cautious and conservative due to concerns that interacting with the video during peak psilocybin effects could be harmful or interfere with the mystical and therapeutic experience. The Intro Video shown at the conclusion of the preparatory session combined with the Opening video viewed at the start of the dosing session were designed to extend the standard pre-dosing preparatory procedures and support the participant as the psilocybin effects began which is a time when participants often experience a surge of anxiety. Psychedelics increase suggestibility (30, 31) and while there is great interest in potentially leveraging this suggestibility to improve therapeutic outcomes by delivering interventions, such as cognitive/behavioral therapies or video/virtual reality applications, during the theorized post-psychedelic “window of neuroplasticity” research in this area should proceed cautiously. There is potential for harm if suggestions have unintended negative consequences as well as ethical concerns around intervening when participants are particularly vulnerable. The Closing Video was designed to convey a sense of calm, safety, and welcome to participants as peak psilocybin effects resolved and was optional so that participants and clinicians could decline viewing the video if it seemed inappropriate based on the participants status at the time in the session. Unfortunately, we did not collect data regarding the reasons or circumstances for cases where the Closing Video was declined and this would be interesting to explore in future studies. Furthermore, there was no sign that the Closing Video had an impact, positive or negative, on post-session alcohol use outcomes which is not surprising as uptake of the Closing Video was variable and the content of the video was not alcohol-related. Whether video interventions at other time points before, during, and after the psychedelic session would be safe and beneficial is not known but warrants further exploration in future studies as well. The Visual Healing intervention contained specific nature-based material created by a world-class filmmaker (LS) and whether our results can be generalized to other video interventions in psychedelic therapy or are specific to the content of the nature videos used here is unknown. A variety of nature settings tailored to the preferences of the patient would be very interesting to explore although the cost of producing multiple videos may be prohibitive. Measures of nature connectedness were collected from participants during the trial but results of analyses of these measures will be reported elsewhere for brevity and clarity purposes. Although the current study was unable to assess whether specific thematic, visual, musical, or cinematic aspects of the Visual Healing intervention may have contributed to the findings we are intrigued by this potential line of inquiry and hope that future research will explore these variables.

## 5. Conclusion

Incorporation of the Visual Healing intervention within open-label psilocybin-assisted therapy for AUD was feasible, safe, and well-tolerated in this pilot randomized, controlled trial. Preliminary findings suggest that Visual Healing has potential to reduce the cardiovascular risks of psychedelic therapy, assist patients in feeling more relaxed and prepared for the psychedelic session, and improve patient satisfaction without interfering with the psychedelic/mystical experience or alcohol-related treatment outcomes. In general, these findings reinforce the importance of set and setting in psychedelic-assisted therapy and in particular highlight the influence of set and setting on safety-related outcomes. More specifically, our study suggests that interventions aimed at optimizing, augmenting, or enhancing set and setting, especially nature-themed immersive video such as Visual Healing, is a promising approach to designing the safest and most effective models of psychedelic therapy. Studies to replicate our findings with Visual Healing in psilocybin-assisted therapy for AUD as well as studies of different set and setting interventions with other psychedelic medications and indications are warranted.

## Data availability statement

The raw data supporting the conclusions of this article will be made available by the authors, without undue reservation.

## Ethics statement

The studies involving human participants were reviewed and approved by Providence IRB. The patients/participants provided their written informed consent to participate in this study. The studies were conducted in accordance with the local legislation and institutional requirements. The participants provided their written informed consent to participate in this study.

## Author contributions

KH, LS, and DK contributed to the conception and design of the study. KH wrote the protocol and oversaw all aspects of study operations and wrote the first draft of the manuscript. LS created the videos for the study with input from KH and DK regarding what would be most appropriate for the study. RR, BY, IB, and JB performed data collection, data cleaning, data analyses, and prepared tables and figures. KS and ML contributed to design and implementation of study therapies. PS performed statistical and data analyses and contributed to manuscript writing. All authors contributed to the article and approved the submitted version.

## Funding

The study was funded by donations from individual donors as well as the Annenberg Foundation to the Pacific Neuroscience Institute Foundation and support from the Saint John’s Health Center Foundation.

## Conflict of interest

KH, DK, and LS are inventors for a provisional patent application by Pacific Neuroscience Institute Foundation for a video-based set and setting intervention. KH, KS, ML, and DK receive clinical trial support from Usona Institute. DK has stock in MindMed, Numinus, and Noetic Fund. KH is a consultant for MindMed. KS is a consultant for Field Trip Health and has stock in Compass Pathways, Field Trip Health, and MindMed. Author LS was employed by company Moving Art.

The remaining authors declare that the research was conducted in the absence of any commercial or financial relationships that could be construed as a potential conflict of interest.

## Publisher’s note

All claims expressed in this article are solely those of the authors and do not necessarily represent those of their affiliated organizations, or those of the publisher, the editors and the reviewers. Any product that may be evaluated in this article, or claim that may be made by its manufacturer, is not guaranteed or endorsed by the publisher.

## Footnotes

^1^ www.pacifictrip.org

^2^ https://www.uclahealth.org/programs/marc/free-guided-meditations/guided-meditations

## References

[1] Schenberg EE. Psychedelic-assisted psychotherapy: a paradigm shift in psychiatric research and development. Front Pharmacol. (2018) 9:733. doi: 10.3389/fphar.2018.00733 30026698 PMC6041963

[2] Dyck E. Flashback: psychiatric experimentation with LSD in historical perspective. Can J Psychiatry. (2005) 50:381–8. doi: 10.1177/070674370505000703 16086535

[3] Hall W Farrell M. What can we learn from the history of research on psychedelic drugs in the addictions? Addiction. (2021) 116:2936–8. doi: 10.1111/ADD.15560 34382252

[4] Krebs TS Johansen PØ. Lysergic acid diethylamide (LSD) for alcoholism: meta-analysis of randomized controlled trials. J Psychopharmacol. (2012) 26:994–1002. doi: 10.1177/0269881112439253 22406913

[5] Bogenschutz MP Ross S Bhatt S Baron T Forcehimes AA Laska E. Percentage of heavy drinking days following psilocybin-assisted psychotherapy vs placebo in the treatment of adult patients with alcohol use disorder: a randomized clinical trial. JAMA Psychiat. (2022) 79:953–62. doi: 10.1001/JAMAPSYCHIATRY.2022.2096 PMC9403854 36001306

[6] Hartogsohn I. Constructing drug effects: a history of set and setting. Drug Sci Policy Law. (2017) 3:205032451668332. doi: 10.1177/2050324516683325

[7] Simonsson O Hendricks PS Chambers R Osika W Goldberg SB. Prevalence and associations of challenging, difficult or distressing experiences using classic psychedelics. J Affect Disord. (2023) 326:105–10. doi: 10.1016/J.JAD.2023.01.073 36720405 PMC9974873

[8] Haijen ECHM Kaelen M Roseman L Timmermann C Kettner H Russ S. Predicting responses to psychedelics: a prospective study. Front Pharmacol. (2018) 9:897. doi: 10.3389/fphar.2018.00897 30450045 PMC6225734

[9] Ko K Knight G Rucker JJ Cleare AJ. Psychedelics, Mystical experience, and therapeutic efficacy: a systematic review. Front Psych. (2022) 13:917199. doi: 10.3389/FPSYT.2022.917199 PMC9340494 35923458

[10] Nikolaidis A Lancelotta R Gukasyan N Griffiths RR Barrett FS Davis AK. Subtypes of the psychedelic experience have reproducible and predictable effects on depression and anxiety symptoms. J Affect Disord. (2023) 324:239–49. doi: 10.1016/J.JAD.2022.12.042 36584715 PMC9887654

[11] Sekula AD Downey L Puspanathan P. Virtual reality as a moderator of psychedelic-assisted psychotherapy. Front Psychol. (2022) 13:813746. doi: 10.3389/FPSYG.2022.813746 35310225 PMC8931418

[12] Masters REL Jean H. The varieties of psychedelic experience: the classic guide to the effects of LSD on the human psyche. Rochester, VT: Park Street Press (2000). 326 p.

[13] Watts R Kettner H Geerts D Gandy S Kartner L Mertens L. The Watts connectedness scale: a new scale for measuring a sense of connectedness to self, others, and world. Psychopharmacology. (2022) 239:3461–83. doi: 10.1007/S00213-022-06187-5 35939083 PMC9358368

[14] Watts R Day C Krzanowski J Nutt D Carhart-Harris R. Patients’ accounts of increased “connectedness” and “acceptance” after psilocybin for treatment-resistant depression. J Humanist Psychol. (2017) 57:520–64. doi: 10.1177/0022167817709585

[15] Carhart-Harris RL Erritzoe D Haijen E Kaelen M Watts R. Psychedelics and connectedness. Psychopharmacology. (2018) 235:547–50. doi: 10.1007/s00213-017-4701-y 28795211

[16] Gandy S Forstmann M Carhart-Harris RL Timmermann C Luke D Watts R. The potential synergistic effects between psychedelic administration and nature contact for the improvement of mental health. Health Psychol Open. (2020) 7:2055102920978123. doi: 10.1177/2055102920978123/ASSET/IMAGES/LARGE/10.1177_2055102920978123-FIG_1.JPEG 33335742 PMC7724423

[17] Kellert SR. The biological basis for human values of nature. The Biophilia Hypothesis. (1993) 42:69.

[18] Buss DM. The evolution of happiness. Am Psychol. (2000) 55:15. doi: 10.1037/0003-066X.55.1.15 11392858

[19] Pati D Freier P O’Boyle M Amor C Valipoor S. The impact of simulated nature on patient outcomes: a study of photographic sky compositions. Health Environ Res Des J. (2016) 9:36–51. doi: 10.1177/1937586715595505 26199272

[20] Lankston L Cusack P Fremantle C Isles C. Visual art in hospitals: case studies and review of the evidence. J R Soc Med. (2010) 103:490–9. doi: 10.1258/jrsm.2010.100256 21127332 PMC2996524

[21] Diette GB Lechtzin N Haponik E Devrotes A Rubin HR. Distraction therapy with nature sights and sounds reduces pain during flexible bronchoscopy: a complementary approach to routine analgesia. Chest. (2003) 123:941–8. doi: 10.1378/chest.123.3.941 12628899

[22] Whall AL Black ME Groh CJ Yankou DJ Kupferschmid BJ Foster NL. The effect of natural environments upon agitation and aggression in late stage dementia patients. Am J Alzheimers Dis Other Dement. (1997) 12:216–20. doi: 10.1177/153331759701200506

[23] Pettinati HM Weiss RD Dundon W Miller WR Donovan D Ernst DB. A structured approach to medical management: a psychosocial intervention to support pharmacotherapy in the treatment of alcohol dependence. J Stud Alcohol Suppl. (2005) 66:170–8. doi: 10.15288/JSAS.2005.S15.170 16223068

[24] Holze F Vizeli P Ley L Müller F Dolder P Stocker M. Acute dose-dependent effects of lysergic acid diethylamide in a double-blind placebo-controlled study in healthy subjects. Neuropsychopharmacology. (2021) 46:537–44. doi: 10.1038/s41386-020-00883-6 33059356 PMC8027607

[25] Johnston CB Mangini M Grob C Anderson B. The safety and efficacy of psychedelic-assisted therapies for older adults: knowns and unknowns. Am J Geriatr Psychiatry. (2023) 31:44–53. doi: 10.1016/J.JAGP.2022.08.007 36184377

[26] Yip R Swainson J Khullar A McIntyre RS Skoblenick K. Intravenous ketamine for depression: a clinical discussion reconsidering best practices in acute hypertension management. Front Psych. (2022) 13:2218. doi: 10.3389/FPSYT.2022.1017504/BIBTEX PMC9556663 36245888

[27] Calleja-Conde J Morales-García JA Echeverry-Alzate V Bühler KM Giné E López-Moreno JA. Classic psychedelics and alcohol use disorders: a systematic review of human and animal studies. Addict Biol. (2022) 27:27. doi: 10.1111/ADB.13229 PMC9541961 36301215

[28] Grabski M McAndrew A Lawn W Marsh B Raymen L Stevens T. Adjunctive ketamine with relapse prevention-based psychological therapy in the treatment of alcohol use disorder. Am J Psychiatry. (2022) 179:152–62. doi: 10.1176/APPI.AJP.2021.21030277 35012326

[29] Dakwar E Levin F Hart CL Basaraba C Choi J Pavlicova M. A single ketamine infusion combined with motivational enhancement therapy for alcohol use disorder: a randomized midazolam-controlled pilot trial. Am J Psychiatry. (2020) 177:125–33. doi: 10.1176/appi.ajp.2019.19070684 31786934

[30] Hartogsohn I. The meaning-enhancing properties of psychedelics and their mediator role in psychedelic therapy, spirituality, and creativity. Front Neurosci. (2018) 12:129. doi: 10.3389/fnins.2018.00129 29559884 PMC5845636

[31] Carhart-Harris RL Kaelen M Whalley MG Bolstridge M Feilding A Nutt DJ. LSD enhances suggestibility in healthy volunteers. Psychopharmacology. (2015) 232:785–94. doi: 10.1007/S00213-014-3714-Z 25242255

