# Supplementary material for: Nature-themed video intervention may improve cardiovascular safety of psilocybin-assisted therapy for alcohol use disorder
Source: Front Psychiatry. 2023 Sep 18;14:1215972. doi: 10.3389/fpsyt.2023.1215972 (PMC10545868; doi:10.3389/fpsyt.2023.1215972)
Supplement: Supplementary file 1 [file Data_Sheet_1.docx]

**Supplementary Figure Legends**

Supplementary Figure 1: Psychedelic dosing room with participant reclining on couch viewing Visual Healing video attended to by two clinician team (participant portrayed by actor).

Supplementary Figure 2: Participant responses to PROMIS Alcohol Use questions for participants in the Visual Healing (VH) versus Standard (STD) groups by study week (* Week 3 = psilocybin 25 mg plus Visual Healing or Standard procedures by random assignment; † Week 7: psilocybin 25 mg plus Visual Healing or Standard procedures by participant choice).

Supplementary Figure 3: Challenging experiences assessed with the Challenging Experience Questionnaire (CEQ) at the conclusion of the Week 3 psilocybin dosing session among participants randomly assigned to the Visual Healing (N=10) versus Standard group (N=10). * p < 0.05 for Visual Healing versus Standard.

Supplementary Figure 4: (A) Anxiety assessed with the State-Trait Anxiety Inventory (STAI) at study baseline (Week 0), before the Week 2 Prep counseling session (Week 2), and before (Week 3) and one day after (Week 3.1) the Week 3 psilocybin dosing session among participants randomly assigned to the Visual Healing (N=10) versus Standard group (N=10). * p < 0.05 for comparison. (B) Anxiety scores following 25 mg psilocybin in Weeks 3 and 7 for individual participants in the Standard group who chose (N=6) versus did not choose (N=3) to view the Visual Healing video in Week 7.

Supplementary Figure 5: Morning salivary cortisol levels for participants randomly assigned to randomly assigned to the Visual Healing (VH; N=10) versus Standard (Std; N=10) groups measured on the morning of the preparatory counseling session (Week 2), the psilocybin dosing session (pre-psilocybin, Week 3), and the post-psilocybin integration counseling session (Week 3.1). * p < 0.05 for Week 2 versus Week 3.1 in Visual Healing group.
