# Supplementary material for: Nature-themed video intervention may improve cardiovascular safety of psilocybin-assisted therapy for alcohol use disorder
Source: Front Psychiatry. 2023 Sep 18;14:1215972. doi: 10.3389/fpsyt.2023.1215972 (PMC10545868; doi:10.3389/fpsyt.2023.1215972)
Supplement: Supplementary file 2 [file Table_1.docx]

**Music Playlist for the Visual Healing Trial**

Link to playlist on Spotify:

<https://open.spotify.com/playlist/0kkSZXk69mSV9vrsAKiLLl?si=d71cfe99d5f44a8d>

| **Song** | **Artist** | **Time** |
| --- | --- | --- |
| 1/1- Music for Airports | Brian Eno | 0:17:21 |
| Scene by the Brook, Symphony No. 6, Pastoral: II | Beethoven, Hanover Band | 0:11:43 |
| Miroirs: II. Oiseaux tristes | Ravel | 0:04:30 |
| It Must Have Been the Coffee | Kaada | 0:04:42 |
| An Ending (Ascent) | Brian Eno | 0:04:25 |
| Omkaaraaya Namaha | Ravi Shankar | 0:01:52 |
| Lotus Feet | Shakti | 0:04:43 |
| Gaayatri | Ravi Shankar | 0:03:24 |
| Illuminations | Devadip Carlos Santana, Alice Coltrane | 0:04:23 |
| The Magic Place | Julianna Barwick | 0:03:51 |
| Flown | Jullianna Barwick | 0:05:04 |
| Polegnala e Todora (Love Song) | Bulgarian State Television Female Choir | 0:03:37 |
| Opening/Napal Morning, Baraka | Michael Stearns | 0:06:02 |
| Let Us Go Into the House of the Lord/Butterfly Sunday | Harold Budd | 0:06:23 |
| Fur Alina | Arvo Part | 0:10:48 |
| Organics | Michael Stearns (Samsara) | 0:03:03 |
| Earth Seen from Above | Meredith Monk | 0:07:14 |
| An Arc of Doves | Brian Eno, Harold Budd | 0:06:27 |
| The Plateaux of Mirror | Brian Eno, Harold Budd | 0:04:13 |
| Lines Made By Walking | John Luther Adams | 0:10:09 |
| Become Desert | John Luther Adams | 0:40:22 |
| Imaginary Galaxies: Galaxy No.1 | Lawrence Bell | 0:20:13 |
| Melodic Communion | Stuart Dempster | 0:13:10 |
| Cloud Landings | Stuart Dempster | 0:16:04 |
| #20-Selected Ambient Works Volume II (Lichen) | Aphex Twin | 0:04:15 |
| #3- Selected Ambient Works Volume II | Aphex Twin | 0:07:44 |
| Spiegel im Spielgel (Cello, Piano) | Arvo Part | 0:09:13 |
| Guru Bandana (Prayer) | Ali Akbar Khan | 0:05:36 |
| Sakhi | John McLaughlin | 0:08:26 |
| Memory Gongs | Cocteau Twins, Harold Budd | 0:07:27 |
| Ooze Out and Away, Onehow | Cocteau Twins, Harold Budd | 0:03:41 |
| Motion Picture Soundtrack | Radiohead | 0:03:20 |
| Ganesha | Alice Coltrane | 0:02:43 |
| Rosetti Noise/Chrystal Garden and a Coda | Harold Budd | 0:14:20 |
| Song to the Siren | This Mortal Coil | 0:03:31 |
| Let It Be- Long Version (Across the Universe) | Carol Wood, Timothy T. Mitchum | 0:03:46 |
| Somewhere Over the Rainbow/What a Wonderful World | Israel Kamakawiwo'ole | 0:05:08 |
| Betelehemu | Wendell P. Whalum, B Olatunji, BYU Men's Chorus | 0:05:10 |
| Alma | Jonny Greenwood | 0:04:07 |
| Pyramid Song (Arr Lawson) | Radiohead | 0:04:34 |
| Dream Baby | Elliot Goldenthal, Elizabeth Fraser | 0:04:30 |
| Bye | Elliott Smith | 0:01:53 |
| The Last Refugee | Roger Waters | 0:04:13 |
| 7:25 | Mogwai | 0:05:04 |
| Circumradiant Dawn | Dead Can Dance | 0:03:17 |
| Teardrop | Massive Attack | 0:05:31 |
| Passion | Peter Gabriel | 0:07:38 |
| Thursday Afternoon | Brian Eno | 1:00:50 |
