# Supplementary figures and images for: Nature-themed video intervention may improve cardiovascular safety of psilocybin-assisted therapy for alcohol use disorder
Source: Front Psychiatry. 2023 Sep 18;14:1215972. doi: 10.3389/fpsyt.2023.1215972 (PMC10545868; doi:10.3389/fpsyt.2023.1215972)

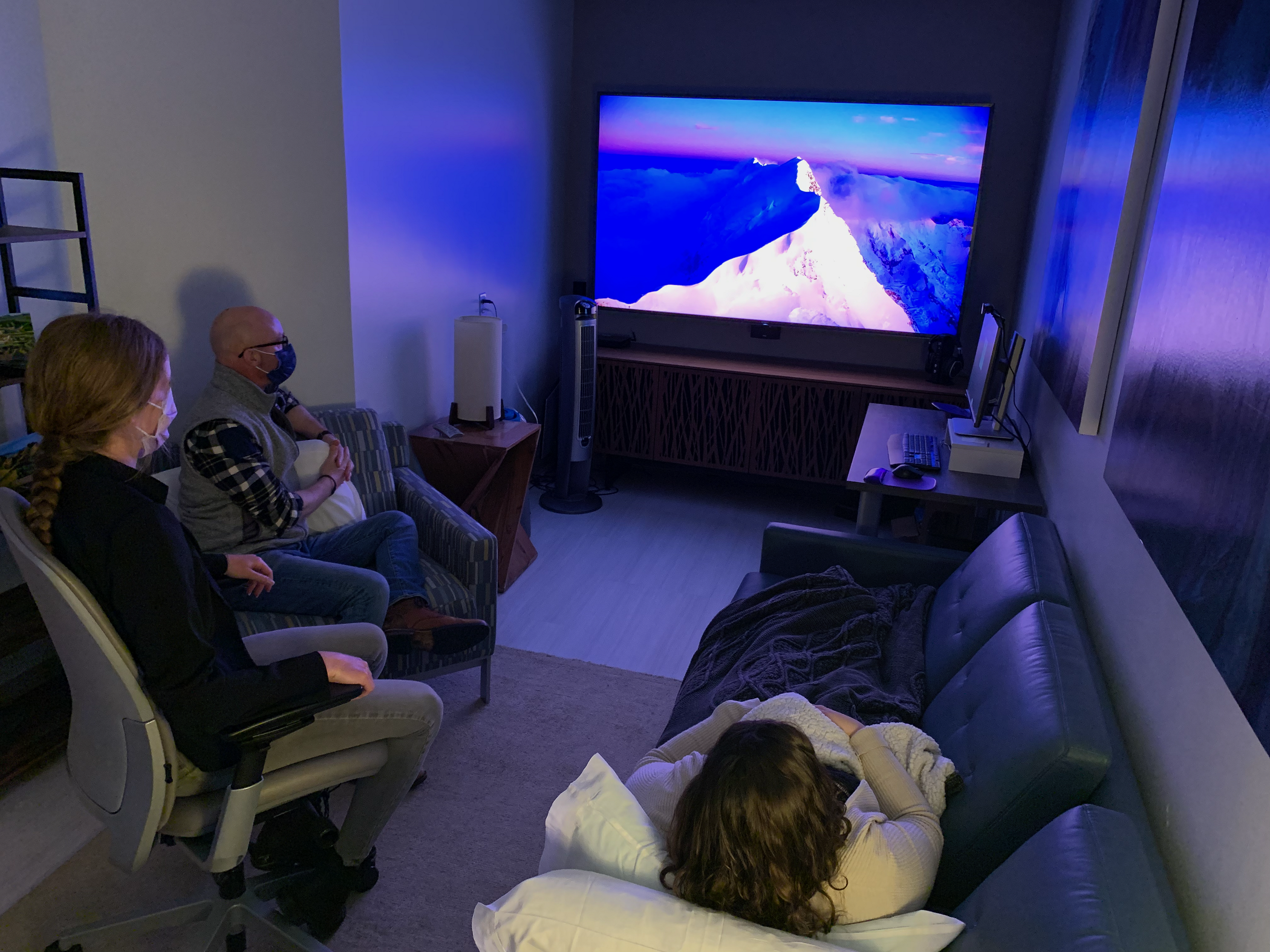

Supplement: Supplementary file 3 [file Image_1.TIFF]

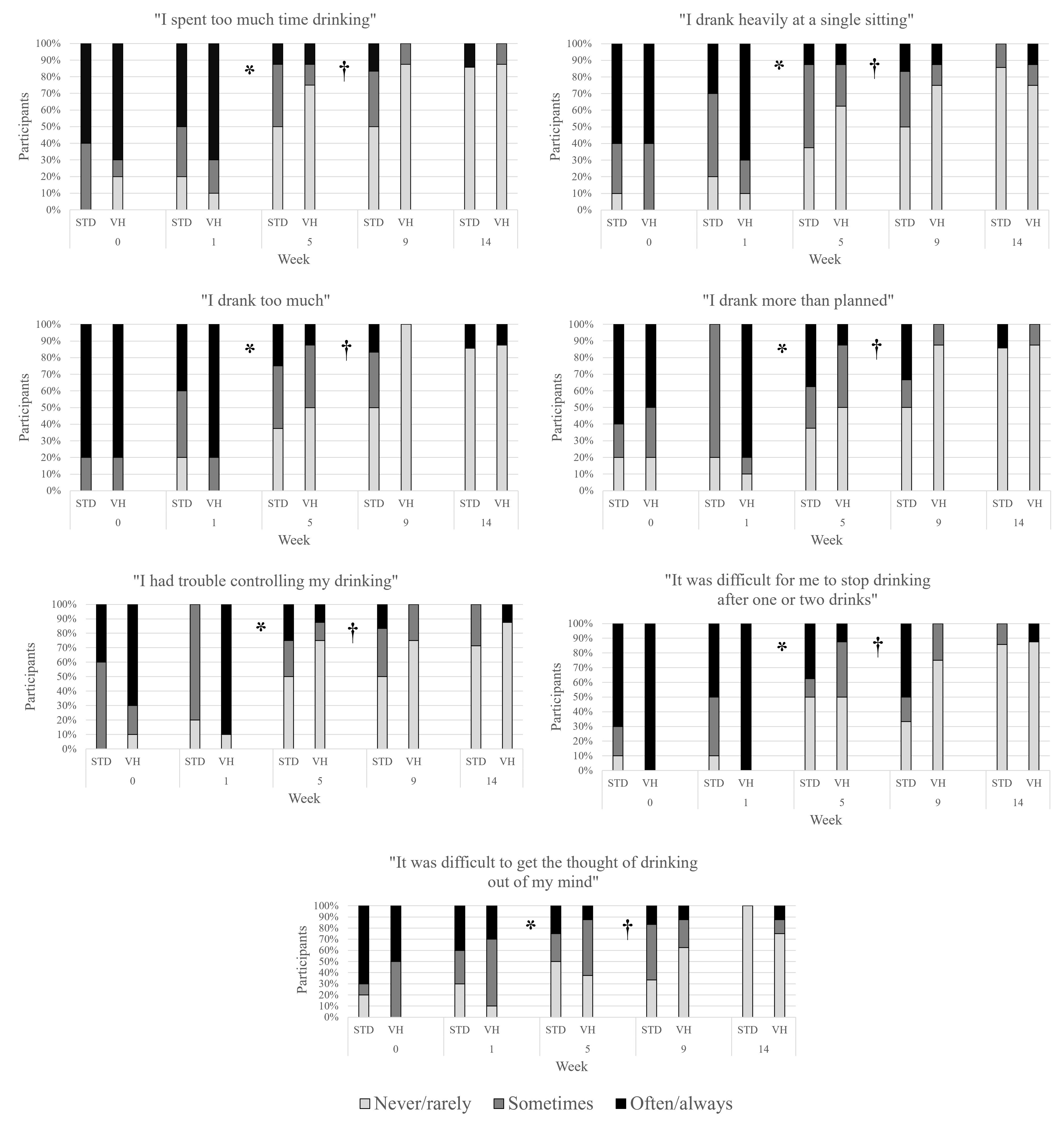

Supplement: Supplementary file 4 [file Image_2.TIF]

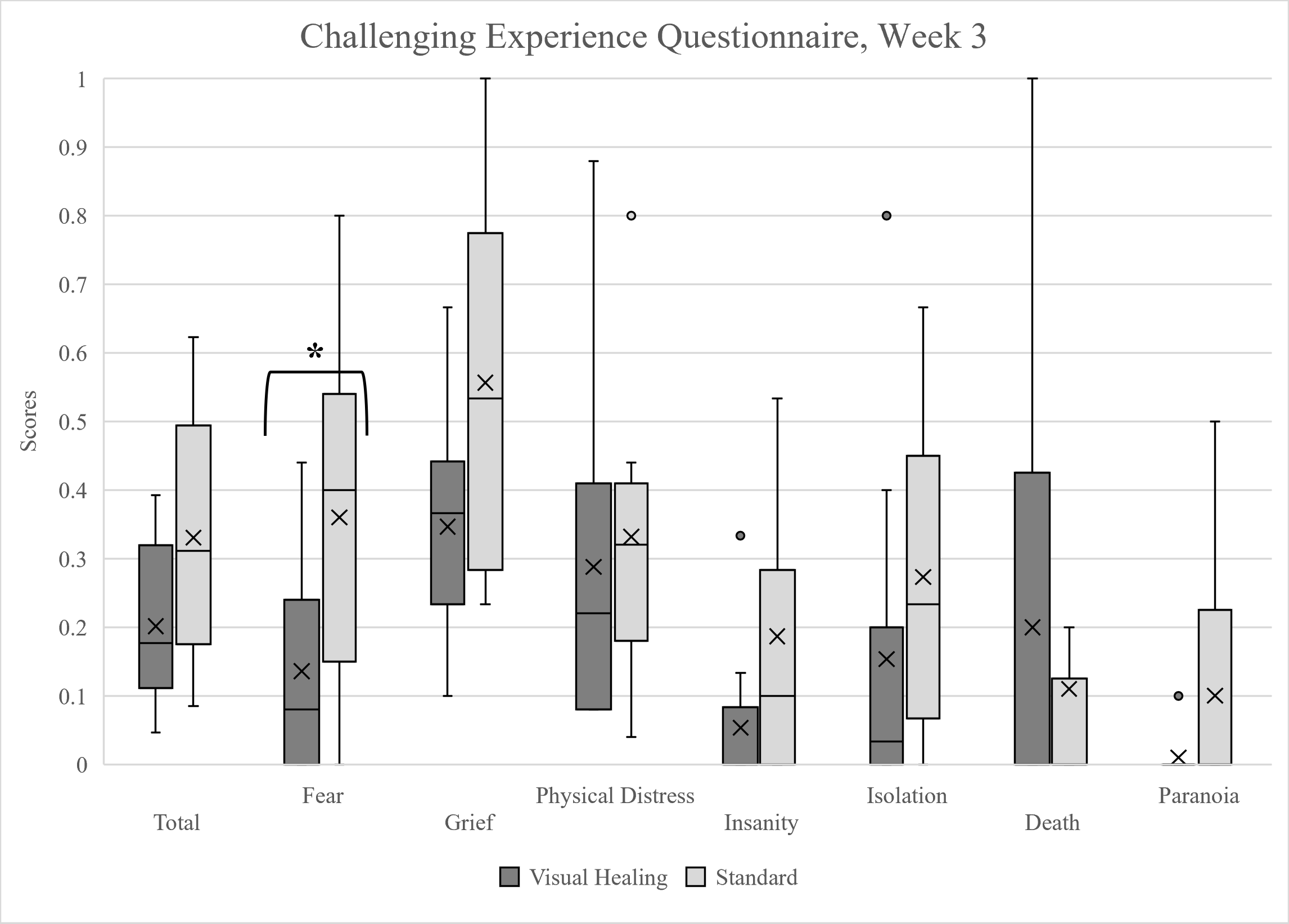

Supplement: Supplementary file 5 [file Image_3.TIF]

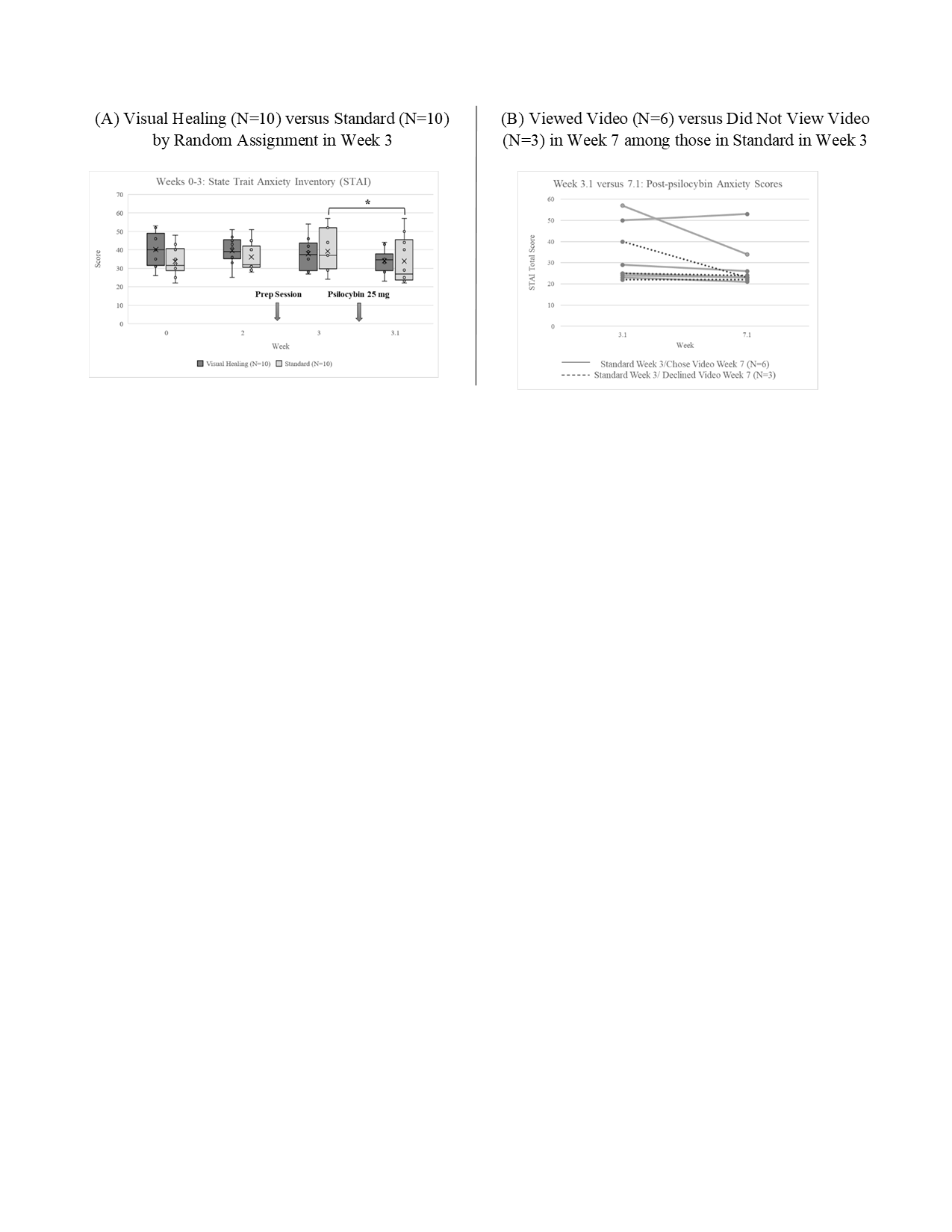

Supplement: Supplementary file 6 [file Image_4.TIF]

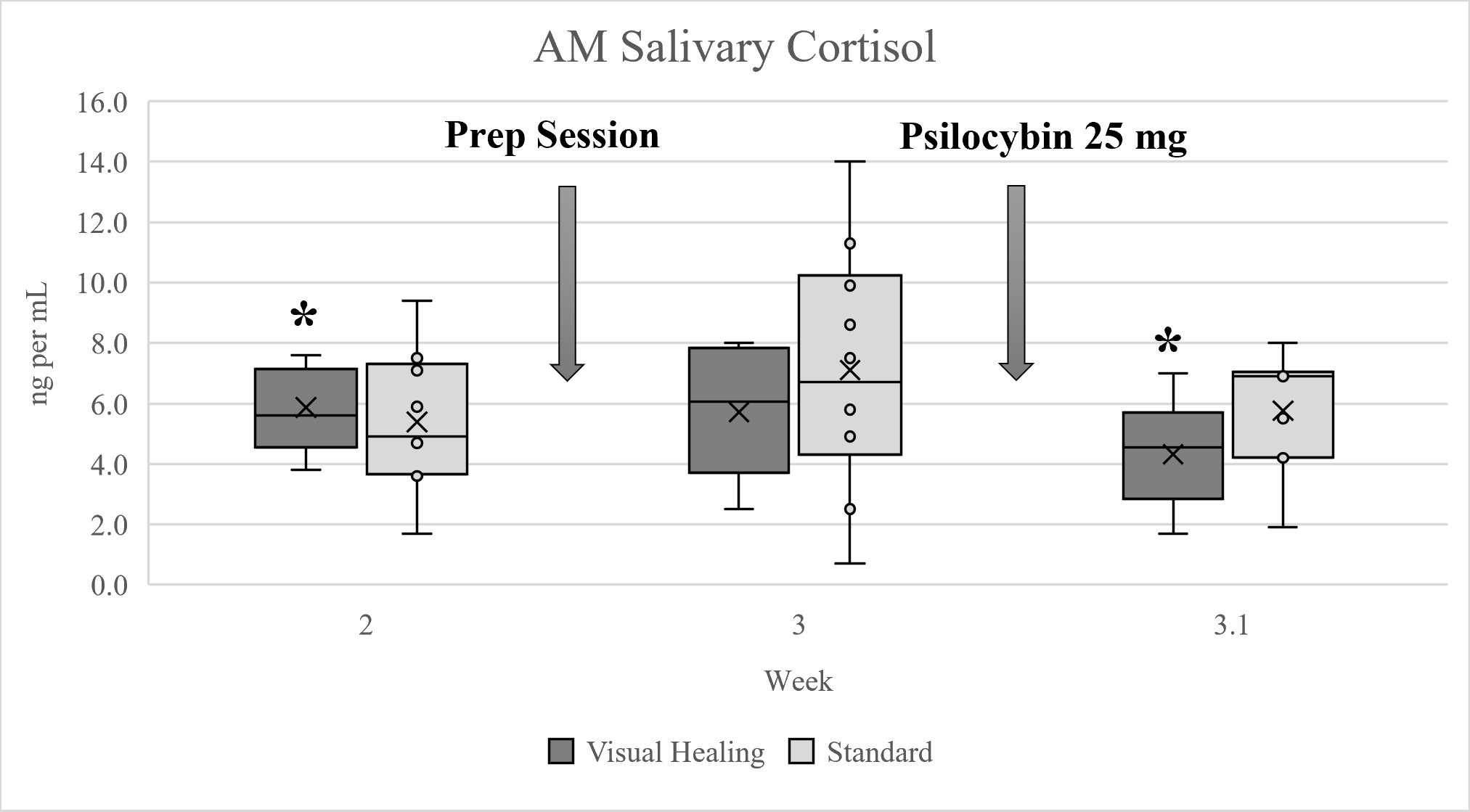

Supplement: Supplementary file 7 [file Image_5.TIF]
